# Plagiarism

**Published:** 1847-06

**Authors:** 


*Plagiarism.*—A volume "*On Tumours of the Uterus and its Appendages*, by Thomas S. Lee," which received the Jacksonian prize, has very recently (1847) been published in London. If any one will look over its pages, and particularly over table No. 11, and the deductions drawn from it, commencing on page 183, and compare it with a similar synopsis, published in the *Amer. Med. Journ.*, for April, 1845, page 330, he will there find a table of the *same* kind, having the *same* arrangement, *similar* headings, and even the *same language*, with full references, by Dr. W. L. Atlee, of this city; yet we cannot discover that Mr. Lee has made any acknowledgment to Dr. Atlee. On the contrary, he assumes the authorship in these words, page 183: "*I have carefully collected into a tabular form all the known operations for the extraction of the ovary!*" We happen to know that the construction of this table cost Dr. Atlee much time, trouble, and labor. His careful synopsis of the leading features of each case, with the references to authorities, was peculiarly valuable to the profession in the consideration of this important question. It is evident, on the pages of Mr. Lee's work, that he has availed himself extensively of this aid, and we regret that he did not think it best to award justice to whom it is strictly due. It would appear from the foot notes, p. 270, that Dr. Atlee's table was before him. The *only original* matter furnished by Mr. Lee is the *addition* of *some* cases occurring since the publication of Dr. Atlee's table, and arranged under the *same* heads. He has failed, however, in noting *all.* So entirely has he depended upon Dr. Atlee's table, that he has not even added four *recent* cases, occurring in *his own country*, and *noticed* in the journals before his work went to press.—*Medical News.*

